# Selenomethionine Incorporation into Amyloid Sequences Regulates Fibrillogenesis and Toxicity

**DOI:** 10.1371/journal.pone.0027999

**Published:** 2011-11-22

**Authors:** Javier Martínez, Silvia Lisa, Rosa Sánchez, Wioleta Kowalczyk, Esther Zurita, Meritxell Teixidó, Ernest Giralt, David Andreu, Jesús Avila, María Gasset

**Affiliations:** 1 Instituto de Química-Física Rocasolano, Consejo Superior de Investigaciones Científicas, Madrid, Spain; 2 Department of Experimental and Health Sciences, Pompeu Fabra University, Barcelona Biomedical Research Park, Barcelona, Spain; 3 Institute for Research in Biomedicine, Barcelona, Spain; 4 Department of Organic Chemistry, University of Barcelona, Barcelona, Spain; 5 Centro de Biologia Molecular Severo Ochoa, Consejo Superior de Investigaciones Científicas-Universidad Autónoma de Madrid, Madrid, Spain; University of Melbourne, Australia

## Abstract

**Background:**

The capacity of a polypeptide chain to engage in an amyloid formation process and cause a conformational disease is contained in its sequence. Some of the sequences undergoing fibrillation contain critical methionine (Met) residues which in vivo can be synthetically substituted by selenomethionine (SeM) and alter their properties.

**Methodology/Principal Findings:**

Using peptide synthesis, biophysical techniques and cell viability determinations we have studied the effect of the substitution of methionine (Met) by selenomethionine (SeM) on the fibrillogenesis and toxic properties of Aβ40 and HuPrP(106–140). We have found that the effects display site-specificity and vary from inhibition of fibrillation and decreased toxicity ([SeM^35^]Aβ40, [SeM^129^]HuPrP(106–140) and [SeM^134^]HuPrP(106–140)), retarded assembly, modulation of polymer shape and retention of toxicity ([SeM^112^]HuPrP(106–140) to absence of effects ([SeM^109^]HuPrP(106–140)).

**Conclusions/Significance:**

This work provides direct evidence that the substitution of Met by SeM in proamyloid sequences has a major impact on their self-assembly and toxic properties, suggesting that the SeM pool can play a major role in dictating the allowance and efficiency of a polypeptide chain to undergo toxic polymerization.

## Introduction

Protein conformational diseases share the occurrence of a basic misfolding event that leads to the accumulation of proteins or fragments thereof as distinct oligomeric self-assemblies with gained toxic functions [1]–[3]. Among the various assemblies, amyloids refer to highly ordered cross β-sheet fibrillar aggregates resulting from tight interfacing of complementary β-sheets [4]–[7]. Despite the regulation by covalent modifications such as proteolytic cleavage, glycosilation and oxidation, among others, the gross information dictating the capacity of a polypeptide chain to form an amyloid is contained in its sequence [4], [5], [8]. Therefore, deciphering the rules for modulating these sequences, their conformation and their self-assembly preferences is fundamental for the design of preventive therapies.

Among the different strategies for modifying protein sequences, the replacement of Met residues by SeM is unique in that it occurs in the absence of changes at the nucleic acid level [9], [10]. Like Met, SeM is an essential amino acid for humans and its availability is strictly related to diet [9], [11], [12]. SeM incorporates non-specifically into proteins in competition with Met [12]. As an organic part of the Se pool, the reduction of its levels has been correlated with an enhanced risk of aging disorders [13]–[15]. In principle, Met substitution by SeM is regarded as a structurally inert change that is exploited for the phasing of macromolecular structures in X-ray crystallography [10]. However, some reports indicate changes in the stability of proteins due to the increased hydrophobicity and distinct oxidation susceptibility of SeM compared to Met [16]–[21]. Thus, changes in the Met/SeM ratio can be considered as a source of transient, metabolic or non-coded mutations and their effect on proteins may vary as a function of residue location.

Of the various amyloid-forming sequences, the amyloid β peptides (Aβ40 and Aβ42) and the PrP(106–140) region are essential components of protein deposits in degenerative dementias and share the presence of regulatory Met residues [22]–[32]. Aβ40 and Aβ42, produced by sequential proteolytic cleavage of the amyloid β-protein precursor (APP) by β- and γ-secretase, accumulate both as extracellular amyloid deposits and synaptic oligomers in Alzheimer disease (AD) [27]. In both Aβ peptides, Met^35^, through the oxidation of its side chain, modulates the oligomerization kinetics, the shape of the final polymer (oligomer vs amyloid fibril) and the neurotoxic function [26], [33]–[37]. In prion protein amyloidoses such as Gerstmann-Straussler-Scheinker syndrome and cerebral amyloid angiopathy, fragments overlapping the 106–140 region of the cellular prion protein (PrP^C^) form the specific amyloid deposits [23], [29], [38]. This sequence (HuPrP) contains four Met residues (Met^109^, Met^112^, Met^129^, Met^134^) flanking either side of the palindromic AGAAAAGA region essential for assembly [38]. Of those, Met^109^ and Met^112^ are not conserved in mammalian PrP sequences and their mutation to Val does not impede fibrillation while it regulates the processing at the α-cleavage site [29], [30], [39]. On the other hand Met^129^ and Met^132^ are polymorphic positions in human and deer, respectively, and their substitution by Val or Leu regulates the disease phenotype and the ability to recognize and amplify exogenous prions [22], [28], [29], [32], [40].

To establish the role of Se intake as related to its specific incorporation as SeM into amyloid forming sequences, we have synthesized Aβ40 and HuPrP(106–140) sequences containing SeM as a replacement for Met. In contrast to the single Met^35^ substitution in Aβ40, the presence of four methionines in HuPrP(106–140) allows to investigate the role of the replacement site on fibril formation. Herein we show that the substitution of Met by SeM in Aβ40 and HuPrP(106–140) regulates both amyloid formation and toxicity. For HuPrP(106–140), the inhibitory effect displays site-specificity, with the activity varying from inhibition of fibrillation and decrease in toxicity ([SeM^35^]Aβ40 and [SeM^129^]HuPrP(106–140)) to accelerated fibrillation and modulation of the polymer shape with retention of toxicity ([SeM^109^]HuPrP(106–140) and [SeM^112^]HuPrP(106–140)).

## Results

### SeM incorporation to amyloid sequences

**Figure 1:**
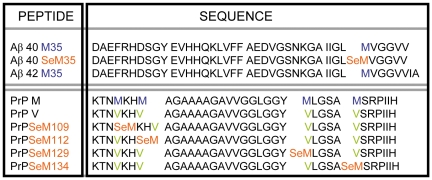
Proamyloid sequences used as templates for the substitution of Met by SeM. Residues are depicted following one-letter code except for selenomethionine that is abbreviated as SeM.

**Figure 1** shows the sequences of Aβ40 and HuPrP(106–140), both with amyloid-forming capacity and with one or more Met residues with relevant roles [27], [29]. The single methionine (Met^35^) in Aβ40 allowed an unambiguous SeM replacement, while the four (Met^109^, Met^112^, Met^129^, Met^134^) in HuPrP(106–140) required a more extensive investigation of the site-specificity of SeM replacement. Hence, the wt HuPrP(106–140) sequence with four SeM residues (all-M), a non-oxidizable variant sequence (all-V) with all four Met residues mutated to Val, plus four analogs with single SeM replacements at either position 109, 112, 129 or 134, and Val at the other positions, were prepared. While all seven peptide sequences are of a size generally regarded as viable for solid phase synthesis methodologies, their well-known tendency to aggregate clearly placed them in the “synthetically difficult” category [41]. This fact, plus the need for cost-effective handling of the high-priced Fmoc-SeM building block, called for highly optimized, state of the art synthetic strategies. Thus, for [SeM^35^]Aβ40, the O-acyl isopeptide approach [42] to difficult sequences was applied, whereby a soluble precursor, 26-O-isoacyl-[SeM^35^]Aβ(1–40), was prepared and purified to near-homogeneity, then incubated at pH 7.4 to give the target peptide in precipitate form. For its part, [SeM^109,112,129,134^]HuPrP(106–140) (all-M), its all-Val counterpart and the four site-specifically SeM-substituted analogs were efficiently assembled by microwave-assisted solid phase synthesis on ChemMatrix®, a resin proven successful in preventing aggregation during the synthesis of large, complex peptides [43]. Peptides were successfully purified from the crude material and their identity and homogeneity confirmed by mass spectrometry [44]. Full details on the synthesis, purification and analytical documentation of all peptides are given in the **Supporting Information S1** file.

### SeM^35^ impedes Aβ40 fibrillation

**Figure 2:**
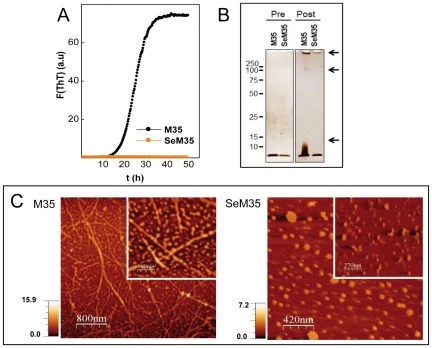
Effect of the incorporation of SeM on the Aβ40 amyloid formation. (**A**) Fibrillation kinetics followed by ThT binding. The displayed curves were obtained by continuous incubation of 20 µM peptide solutions in PBS containing 15 µM ThT at 30°C run in triplicate, and represent the average of three independent experiments. (**B**) Representative silver-stained SDS-PAGE gel of [Met^35^]Aβ40 and [SeM^35^]Aβ40 before (pre) and after 80 h (post) of incubation under aggregating conditions. Arrows indicate the distinct oligomers. (**C**) AFM topography imaging of the aggregation reaction products of [Met^35^]Aβ40 and [SeM^35^]Aβ40 after 80 h of incubation. Inserts displayed the magnification of a representative area of each case.

To investigate the ability of SeM to modify the aggregation properties of Aβ40 we set up a ThT binding kinetics assay. To this end, either wt Aβ40 (Met^35^) or [SeM^35^]Aβ40 were incubated at 20–40 µM concentration in PBS in the presence of 15 µM ThT and the increase in fluorescence emission as consequence of its binding to cross β-sheets was monitored (see methods). **Figure 2A** shows that wt Aβ40 at 20 µM and 30°C provokes a time-dependent increase in ThT fluorescence compatible with the known fibrillation process [45]. Importantly, the kinetic trace was reproducible in independent experiments with different batches, with an average lag-phase of 13±2 h. In contrast, incubation of [SeM^35^]Aβ40 under similar conditions did not cause any detectable change in ThT fluorescence. Increasing [SeM^35^]Aβ40 concentration up to 200 µM and the incubation time up to 1 week did not provoke any significant or additional change. These results suggest that the substitution of Met by SeM has a clear deleterious impact on amyloid formation.

To confirm the previous findings we analyzed the reaction products by Atomic Force Microscopy (AFM). **Figure 2C** shows that, as expected from the ThT fluorescence readings, Aβ40 assembles into long (>200 nm length) and thin (7 nm height and about 2×40 nm diameter) fibrils that appear decorated by globular particles of about 4 nm in height and 40–80 nm in diameter [**Figure 2C** **insert**]. In contrast, [SeM^35^]Aβ40 uniquely yields globular aggregates characterized by 3.5–7 nm height and 35 nm average diameter, corroborating the impairment of the fibrillation process. On the other hand, [SeM^35^]Aβ40 aggregates yielded electrophoretic patterns different from those of toxic Aβ40 oligomers [**Figure 2B**] [46].

### SeM effect on fibrillation displays site-specificity

To ascertain whether the previous findings are uniquely related to the Aβ40 sequence and the essential role played by Met^35^, or can take place also in other sequences, we analyzed the effect of this substitution on the fibrillation properties of HuPrP(106–140). In this case, given the presence of four Met residues and the possible interference of undesired oxidations in long time incubations, we investigated a non-oxidizable version (all-V, all four Met replaced by Val), as well as single SeM replacements with Val at the other positions [**Figure 1**].

**Figure 3:**
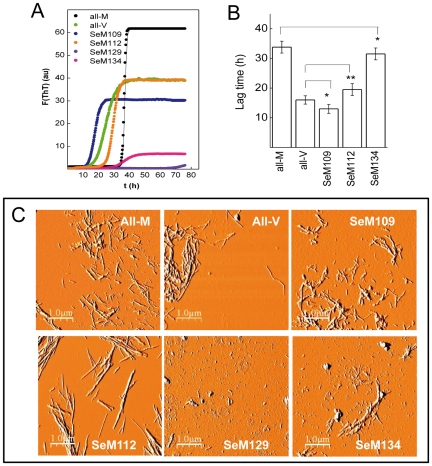
Effect of the incorporation of SeM in HuPrP(106–140) on its amyloid formation. (**A**) Representative fibrillation kinetics of HuPrP(106–140) sequences followed by ThT binding. The color code of the traces is depicted as an insert in each panel. The displayed curves were obtained by continuous incubation of 20 µM peptide solutions in PBS containing 15 µM ThT at 30°C run in triplicate, and represent the average of three independent experiments. (**B**) Lag times of the fibrillation kinetics of HuPrP(106–140) and of its variants. Lag times were calculated independently from each curve and analyzed statistically using the Student's t-test tool provided by Origin 6 software: *, non-significant; **, *P*<0.05. (**C**) Phase images of the molecular species formed after 80 h incubation of HuPrP(106–140) sequences. The displayed fields for all-V, all-M, SeM^109^ and SeM^112^ sequences represent most frequent areas (9 out 10 analyzed 1 µm×1 µm regions). For SeM^129^ and SeM^134^ the displayed fields represent the minor hits (1 out of 20 analyzed 1 µm×1 µm regions).

**Figure 3A** shows that, at 30°C, 20 µM concentration in PBS and with mild orbital shaking, both all-M and all-V HuPrP(106–140) undergo fibrillation, though with notable kinetic differences. Thus, all-M HuPrP(106–140) exhibits the kinetic profile of a highly cooperative process, characterized by an average lag time of 33.8±2.0 h and a final arbitrary ThT fluorescence intensity of 60±5, whereas fibrillation of the all-V variant is characterized by a lag-phase of about 16.4±2.0 h and a final ThT intensity reading of 40±4 [**Figures 3A and 3B**]. These differences found here for the (106–140) sequence regions agree with previous findings reporting the faster polymerization of [Val^129^]PrP compared to [Met^129^]PrP and the higher propensity of [Met^129^]PrP(109–135) over its Val^129^ variant to form β-sheet stabilized fibers [40], [47]. Placing SeM at position 109 slightly reduces both the lag phase and the final ThT intensity of the fibrillation kinetics. However, the absence of a clear statistical significance in these changes suggests that SeM^109^ behaves as an all-Val variant. On the contrary, placing SeM at position 112 significantly increases the lag time to 19.7±1.2 h with no effects in the maximum ThT intensity. Surprisingly, the introduction of SeM at position 129 drastically impairs the fibrillation process. Prolonged incubations (up to 1 week) yielded ThT intensity increases below 2.5 with averaged lag phases of >72 h. Along similar lines but to a lesser extent, placing SeM at position 134 allowed a slight fibrillation process featured by a final ThT intensity of 8 and a lag time of 31.5±2.0 h.

**Figure 4:**
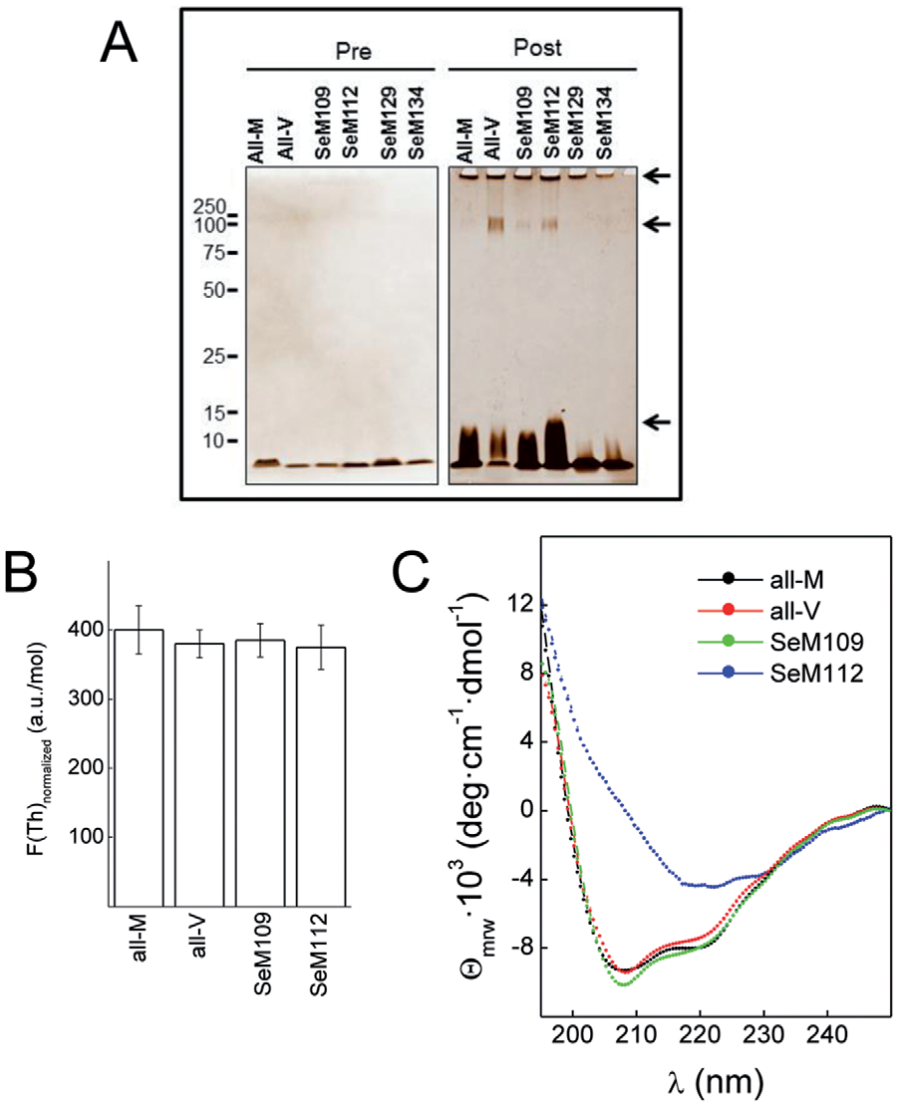
Aggregation profiles of HuPrP(106–140) sequence variants. (**A**) Typical aggregation pattern of HuPrP(106–140) and of its variants probed by silver-stained SDS-PAGE. Peptide aliquots (1 µg) before (pre) and after 80 h (post) of aggregation were separated in TGX-Precast BioRad gels and then silver stained. (**B**) Normalized ThT binding of the insoluble aggregates formed by HuPrP(106–140) peptides. Aggregated peptides were isolated as insoluble pellets of 30 min centrifugations at 15000 rpm, resuspended in PBS at 60 µM concentration. ThT binding of the resuspended aggregates was measured by fluorescence at 20 µM peptide and 15 µM ThT concentrations. (**C**) Far-UV CD spectral features of the insoluble aggregates formed by HuPrP(106–140) peptides. Insoluble pellets were prepared as in panel B in PBS and the spectra recorded at 60 µM. At least three separate experiments were performed to confirm these results.

To confirm these findings we analyzed by AFM the products of the aggregation reactions [**Figure 3C**]. In agreement with the ThT kinetics, the all-M, all-V, SeM^109^ and SeM^112^ versions of HuPrP(106–140) yielded fibrillar structures, of which those formed by SeM^112^ differed notably from the others by appearing as regular straight rods with a high homogeneity in length. On the contrary, the reaction product of the SeM^129^ analog yielded mainly amorphous aggregates with rarely the presence of fibrillar aggregates. The SeM^134^ peptide displayed an intermediate behaviour, showing few but detectable fibrillar assemblies. The aggregation profile was also studied by SDS-PAGE. **Figure 4A** shows that on aggregation all-M, all-V, SeM^109^ and SeM^112^ versions of PrP(106–140) yielded bands of SDS-resistant aggregated species, whereas SeM^129^ and SeM^134^ ran mainly as monomers.

Taken together these data indicate that, as for the case of Aβ40, SeM incorporation also impairs HuPrP(106–140) fibrillation, but in this case the inhibitory process is highly dependent on the replacement site, with position 129 and to a lesser extent 134 being essential in this respect.

### SeM modulates fibril shape

As noted above, [SeM^112^]HuPrP(106–140) forms fibrillar aggregates that differ notably from those obtained from all-M, all-V and SeM^109^ peptides. To gain an insight on the basis of this polymorphism, the fibrils were isolated from the aggregation reactions by centrifugation and, after resuspension, were characterized for their ThT binding on fibril molar basis and by far-UV CD for comparison with previous reports [48]. It must be noted that SeM^129^ and SeM^134^ peptides were not considered in this study given their failure to form fibrils with minimal efficiency.

**Figure 4B** shows that the fibrillar aggregates of all-M, all-V, SeM^109^ and SeM^112^ recovered by centrifugation from the aggregation reactions and resuspended to similar molar concentration yield similar ThT intensity, and the minor variations are not statistically significant. **Figure 4C** shows that the all-M, all-V and SeM^109^ peptides shared a common spectrum, featured by a double minimum at 208 and 220 nm suggesting an altered β-sheet structure. On the contrary, the spectrum of the SeM^112^ analog displayed the features of a pure β-sheet structure. The results thus support that, depending on its incorporation site, SeM can modulate the secondary structure and subsequently sculpture its self-assembly shape.

Interestingly, these distinct spectral features have been previously reported for the R- and S-fibrils formed by the full length PrP resulting from the aggregation using two different conditions [48]. In fact the morphological and spectral features herein found for the aggregates formed by the SeM^112^ analog resemble those described for S-fibrils, which can be formed with the HaPrP but not with the MoPrP [48], [49]. Similarly, the features of the fibrils formed by the all-M, all-V and SeM^109^ peptides resemble the properties described for the R-fibrils which can be formed by both HaPrP and MoPrP [48], [49]. Among other sequence differences, HaPrP and MoPrP differ in the residue at position 112, Met in HaPrP and Val in MoPrP. Since Met though not Val can be metabolically replaced by SeM, it is tempting to speculate that the formation of S-fibrils could be dictated at least in part by the presence of chains containing SeM in position 112, which can uniquely occur with HaPrP, not with MoPrP.

### SeM effects on fibrillation are unrelated to oxidation

**Figure 5:**
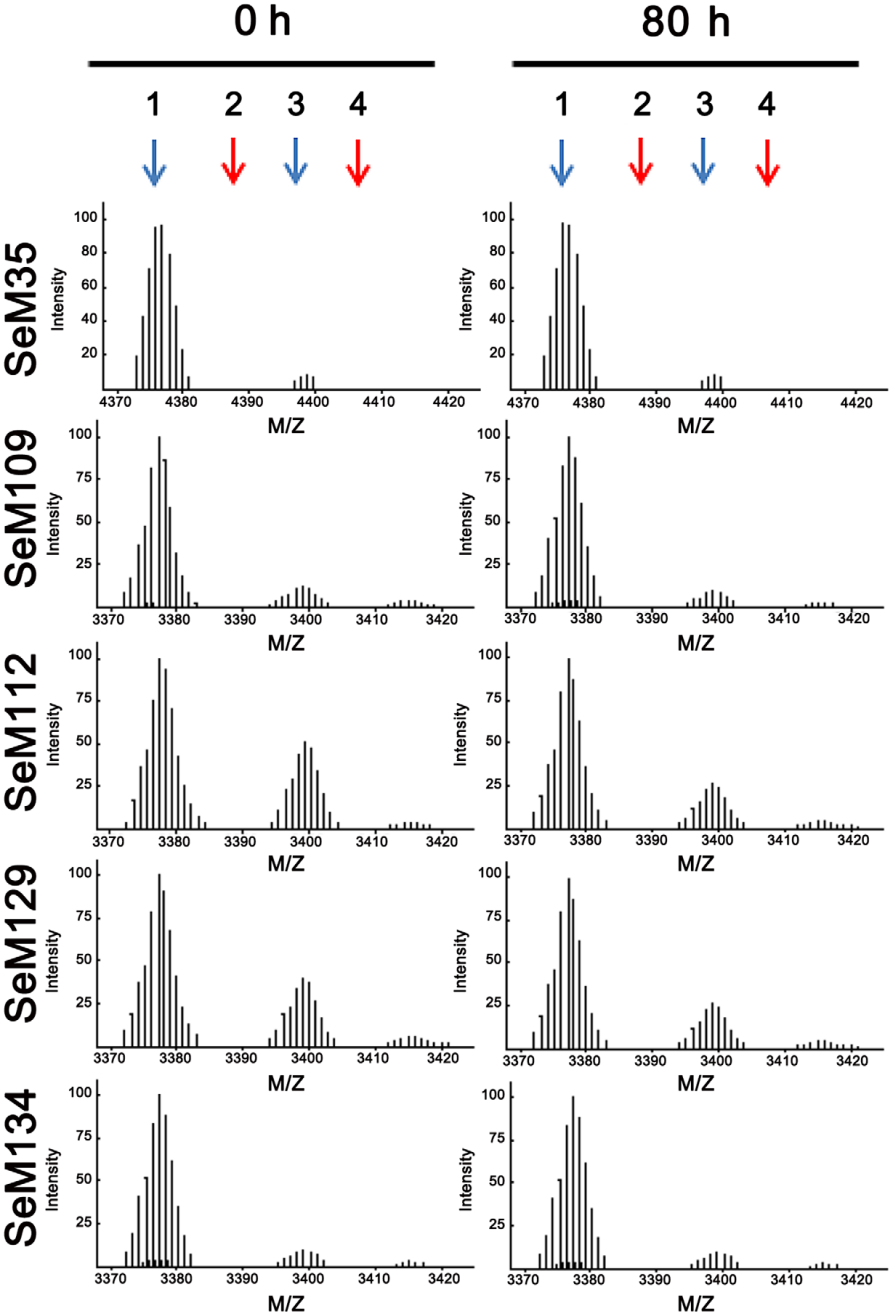
MALDI FT-ICR profiles of SeM-containing Aβ40 and HuPrP(106–140) peptides. Representative m/z patterns of the distinct peptides after 80 h of incubation under aggregating conditions. Measurements were performed using samples of at least two separate experiments. Arrows indicate the theoretical positions for the m/z values of: 1) +1, 2) +16 (selenoxide), 3) +23 (Na+-adduct) and 4) +32 (selenone). For SeM35 the values are: 4376.1, 4381.1, 4388.1, 4408.1. For SeM analogs of HuPrP(106–140) the values are: 3377.2, 3393.2, 3399.2, 3409,2. The peak complexity arises from the Se isotopic distribution [20].

As with Met, the SeM side chain can undergo oxidation to selenoxide and selenone, and if so could drastically modify the process of amyloid formation [26], [33]–[37]. To investigate whether the observed differences in fibrillation were related to SeM oxidation we analyzed by MALDI FT-ICR the peptides before and after the aggregation reaction. In all cases, m/z patterns of pre-aggregated and post-aggregated samples were superimposable and peaks reproduced the theoretical predicted charged masses [**Figure 5** **, Supporting Information**]. Importantly, no peaks at +16/+32 Da expected for the oxidized variants were detected [**Figure 5**]. Despite the non-quantitative nature of the mass spectrometry method, it plausible discards that undesired side chain oxidations play a role in the amyloid formation traits.

### SeM containing sequences also function as exogenous fibrillation regulators

**Figure 6:**
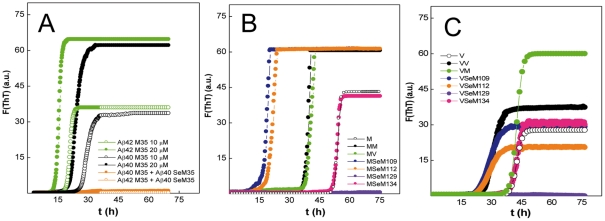
Analysis of the regulatory cross-talk between SeM-tagged and wt sequences. (**A**) ThT binding kinetics of Aβ40 and Aβ42 in the absence (10 and 20 µM) and presence of [SeM^35^]Aβ40 (10 µM of each peptide). (**B**) Time evolution of the ThT binding of mixtures of 10 µM all-M HuPrP(106–126) in the absence and presence of 10 µM of HuPrP(106–126) sequence variants. (**C**) ThT binding kinetics of mixtures of 10 µM all-M HuPrP(106–126) in the absence and presence of 10 µM of HuPrP 106–126 sequence variants. The color code of the different traces is indicated at the right hand side of each panel. The displayed curves were obtained by continuous incubation of the different peptide solutions in PBS containing 15 µM ThT at 30°C in duplicate, and represent the average of three independent experiments.

Consistent with the previous findings and with the fact that Met substitution by SeM would be hardly ever quantitative under physiological conditions, we next tested the capacity of the SeM-containing sequences to modulate the amyloid formation process of the unlabelled sequences. As amyloid formation can be essentially view as a seeded-polymerization in which nucleation, elongation and polymer fragmentation are critical steps, the SeM effects could also provide mechanistic information [1]–[7]. The results are shown in **Figure 6**.

Co-incubation of [SeM^35^]Aβ40 with Aβ40 and with its longer and more fibrillogenic form Aβ42 impaired their fibrillation process [**Figure 6A**]. These impairments could not be attributed to dilution since, in the absence of [SeM^35^]Aβ40 and at equal concentration, both Aβ40 and Aβ42 undergo fibrillation. Rather, the results agreed with an inhibition process and suggested that [SeM^35^]Aβ40 interacts with Met^35^-bearing peptides, and halts their productive aggregation through the formation of growth-impaired oligomers.

Similarly, in the HuPrP(106–140) case, co-incubation of 10 µM SeM^129^ analog with 10 µM of either all-M [**Figure 6B**] or all-V [**Figure 6C**] peptides inhibited amyloid formation. Again, such inhibitions could not be attributed to dilution effects, since the latter peptides, at 10 µM and in the absence of the SeM^129^ analog, yielded ThT binding kinetics compatible with fibrillation reactions. Hence, the inhibition trend suggests that [SeM^129^]HuPrP(106–140), acting like a quencher, interacts with either all-M or all-V HuPrP(106–140), giving rise to oligoheteromeric species that do not sustain growth. This data agreed with previous findings indicating the essential role of identity in position 129 for the allowance of formation of a stable steric zipper [50].

On the contrary, [SeM^134^]HuPrP(106–140), of very low efficiency in fibrillogenesis, when mixed with either all-M or all-V does not alter significantly the ThT binding pattern of the previous peptides [**Figure 6B and 6C**]. These results suggest that SeM^134^ precludes stable interaction and therefore causes its segregation. Since position 134 has not been found to play a fundamental role in amyloid formation, then the segregating behavior seems related to SeM hydrophobic properties and their provoked reactions [5], [51], [52].

On the other hand, SeM^109^ and SeM^112^ when mixed 1∶1 with either all-V or all-M peptides altered the ThT binding kinetics, imposing their characteristic lag-phase and allowing the final ThT intensity of the SeM-free peptide [**Figure 6B and 6C**]. This observation strongly suggests that positions 109 and 112 determine the efficiency of seed formation and therefore the speed of the polymerization reaction [1]–[7].

### SeM-containing sequences can ameliorate toxicity

**Figure 7:**
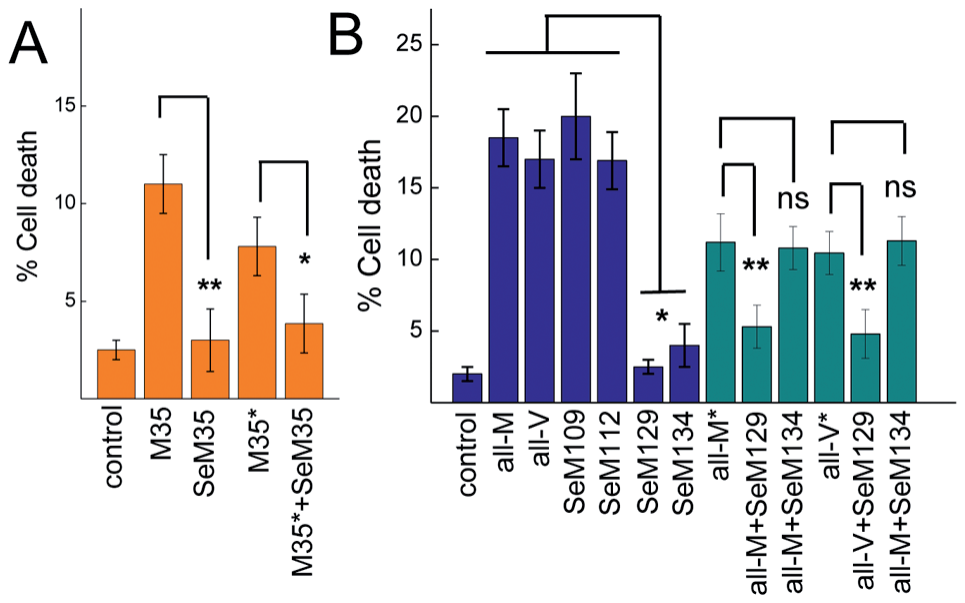
Cytotoxic potential of SeM containing sequences and of their mixtures. Rodent primary cortical neurons were cultured for 7 days on poly-D-lysine-coated coverslips and treated with 10 µM of each peptide or 1∶1 molar ratio mixture of peptides for 48 h. The cells were then probed with LIVE/DEAD kit. The percentage of dead cells was obtained dividing the number of dead cells by the total (live and dead) number of cells. The results are the means ± SD of three independent experiments ran in duplicate. Statistical analysis was performed with the Student's *t* test tool of Origin software. ns, non-significant. *, *P*<0.05; **, *P*<0.005.

To investigate the structure–activity relationship of the SeM substitutions we analyzed the effect of the aggregation reaction products on the viability of rodent primary cortical neurons [**Figure 7**]. For this purposes, the different peptides and their combination were incubated for 80 h at 30°C at 0.10–0.15 mM in PBS and then diluted to a final concentration of 10 µM in the cell medium and allowed to incubate for 48 h. Under the assay conditions, all peptides except [SeM^35^]Aβ40 and the SeM^129^ and SeM^134^-analogs of HuPrP(106–140) and their mixtures have completed their fibrillation process as judged by parallel ThT reading, and the untreated cells yielded cell viability values that amounted to 97.5±1%.

Aβ40 was found to cause 11±1% cell death, in agreement with previous reports [27], [37], [53]. Interestingly, [SeM^35^]Aβ40 reduced cell death to 3±1.5%, thereby excluding any relationship between its assemblies and the highly neurotoxic nonfibrillar oligomers formed by Aβ peptides [27]. This reduction pattern was maintained for its 1∶1 mixture with Aβ40, which cannot be explained solely on the basis of [Met^35^]Aβ40 dilution as judged from the concentration control.

As for HuPrP(106–140), the aggregation reaction products of all-M and all-V caused about 20±2% of cell death, in agreement with the toxicity levels described for the polymers formed by HuPrP(82–146) [54]. SeM^109^ and SeM^112^ analogs caused cell death to a similar extent, in agreement with their similar amyloid forming ability at long incubation times [**Figure 3A**]. On the contrary, SeM^129^ and to a lesser extent SeM^134^ caused minor effects on cell viability (1.1±0.5 and 4±0.6, respectively). As in the case of [SeM^35^]Aβ40, these statistically significant reductions in the extent of cell death compared to that caused by the amyloid-assembled sequences discards active oligomeric species. Moreover, the aggregation products of the all-M and all-V peptides mixed with SeM^129^ and SeM^134^ reproduced the profiles observed in kinetic experiments [**Figure 6B and 6C**]. Mixing SeM^129^ 1∶1 with either all-M or all-V decreases cell death extent to almost abrogation, and the effect cannot be explained solely in terms of the reduction all-M and all-V concentration as shown by the concentration controls. On the contrary, mixing the SeM^134^ analog 1∶1 with either all-M or all-V reproduces the cell death percentage of diluted all-M and all-V peptides.

## Discussion

**Figure 8:**
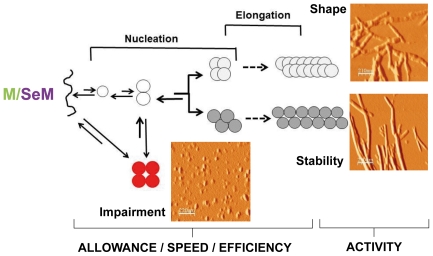
Summary of the effects of SeM introduction in amyloid forming sequences. Replacement of methionine (M) residues by its metabolic competitor selenomethionine (SeM) in proamyloid sequences involves changes in local hydrophobicity and steric factors. With a site-dependence, the replacement can promote side association reaction that either decrease the efficiency and speed or impair amyloid formation. In other cases, by regulating the seed packing can generate distinct fibrilar assemblies.

Unveiling the ways proamyloid sequences can be modulated to impede their productive engagement into self-assembly processes yielding toxic events is essential for designing preventing strategies for conformational diseases. The pioneer study of Goldschmidt et al [5] has solidly established as general principle that the capacity of a protein to form the β-sheet based fibrillar amyloid structures is coded in its sequence, although its display may depend on structural and environmental regulatory factors [55]. One possible modulatory event is that involving metabolic changes of Met and SeM pools and consequently of their competitive incorporation in proteins through the AUG codon. Taking the advantage of synthetic approaches we have substituted Met by SeM in amyloid forming sequences and we have found dramatic effects on their polymerization and toxicity. These effects varied from inhibition ([SeM^35^]Aβ40 and [SeM^129^]PrP(106–140)), polymerization kinetics perturbation to polymer shape determination ([SeM^112^]PrP(106–140)) [**Figure 8**].

Despite the consideration of Met and SeM as structurally equivalent, the change of a sulfur by a selenium atom involves major steric and reactivity differences. Se is slightly larger than S (atomic radius of 1.17 vs 1.04 Å) and has also a larger van der Waals radius (1.90 vs 1.80 Å).Since the spines of amyloid fibrils consist in steric zippers formed by the interdigitation of β-sheets through their side chains, any steric perturbation may lead to clashes which may reduce the stability of this unit or even preclude its formation [5], [50]. This might be the case of the fibrillation impairments of [SeM^35^]Aβ40 and [SeM^129^]PrP(106–140) peptides, for which the crystal structure of shorter fragments have shown Met^35^ and Met^129^ actively participating in the inter-sheet packing [50], [56]. Also, since the interdigitations are not unique but can involve distinct patterns, the side chain size increase together with its position can dictate the preference for specific stacking patterns over others that as seeds will produce distinct fibril shapes as for SeM^109^ and SeM^112^ analogs of PrP(106–140) [5], [50], [56]. The larger size of Se than S also causes SeM to have a larger surface area and hence hydrophobicity than Met. Since amyloid formation is a complex process involving the construction of oligomeric species undergoing growth, fragmentation and quenching or arrest, minor changes in hydrophobicity may trigger significant alterations in the solubility of the distinct oligomeric species as well as in the the features of the interacting surfaces [1]–[7], [50]. For instance, by its increased hydrophobicity SeM can decrease the efficiency of amyloid formation in [SeM^134^]PrP(106–140), which retains the ability to engage in the process but does it with a very low efficiency.

SeM also differs from Met in its side chain oxidation process. SeM can undergo oxidation by peroxynitrites to selenoxide but, unlike the sulfoxide, the selenoxide is easily reduced by organic thiols as glutathione and does not required enzymatic assistance [20], [52], [57]. Although SeM oxidation is not a major event in our experimental setup, since sulfoxide formation is known to impair fibrillation in Aβ40 and PrP(106–126), the chemical differences of the reaction could add novel regulatory steps to the polymerization [24]–[26], [34], [35], [58]. To address this possibility, improved basic knowledge is required on reaction conditions and product characterization of SeM oxidation as a part of a protein and free in solution [52], [57].

For Aβ40 the Met35SeM non-coded or metabolic mutation impairs amyloid formation but stabilizes oligomeric assemblies as shown by AFM. Based on shape considerations, the oligomers might be suspected to act as the actual neurotoxins. However, both PAGE-SDS analysis and toxicity evaluation discard such assemblies being deleterious and support the importance of the amyloid pathway as a source of toxic species. This rationale can be extended to the [SeM^129^]HuPrP(106–140) analog, which in addition to its impaired fibrillation and lack of toxic activity, prevents the fibrillation and toxicity of all-V and all-M. For these two cases, the incorporation of SeM into an essential position functions as a physiological anti-amyloid metabolic defense. However, the effect of SeM incorporation is not homogeneous. For instance, the SeM^109^ and SeM^112^ analogs of HuPrP(106–140) displayed differences in kinetics and in fibril shape, and such morphological differences can have important functional implications [59]. In this sense, fibrils of the SeM^112^ –shape are expected to be more toxic that fibrils with shapes of the SeM^109^-analog, whereas if fragmented the toxicity profile inverts [59]. In our set up, both assemblies yielded statistical similar toxicity traits suggesting that SeM substitution could also play a role in the in vivo stability (fragmentation or recycling) of the polymers so activity differences in the 48 h assay become averaged. In this line, H/D exchange experiments have shown that isolated fibrils can display significant distinct recycling properties, with changes in fibril dissolution rate constant of about two orders of magnitude ( 0.6 s^−1^ and 1.0×10^−2^ s^−1^ for Aβ40 and Aβ42, respectively) [60].

As summarized in **Figure 8**, these evidences clearly indicate that SeM incorporation into pro-amyloid sequences results in various effects as a function of its location and suggest that metabolic changes in the Met/SeM pool can exert important modulatory effects in amyloid diseases.

## Materials and Methods

### Peptides and aggregation reactions

The SeM-substituted versions of Aβ40 and HuPrP(106–140) (Figure 1A) were synthesized by solid phase methods, purified by HPLC and characterized by mass spectrometry. Details are given in the **Supporting Information S1** file. For control studies, Aβ40 and Aβ42 were obtained from GenScript. Lyophilized peptide stocks were dissolved in HFIP, aliquoted and dried under N_2_ for storage at −80°C. Samples were reconstituted in 5 mM NH_4_OH pH 8.0 at about 2 mg/ml concentration and filtered through 0.2 µm membranes before use. Peptide concentrations were determined by UV spectroscopy and by amino acid analysis. Peptide stock solutions were diluted with PBS at 100–200 µM concentrations and kept at 4°C for less than 30 min. The aggregation reactions were performed both in eppendorf tubes and in wells of a 96-well plate by incubating 20–200 µM peptide monomers in PBS at 30°C with orbital shaking (100 rpm).

### Thioflavin T binding kinetics

The kinetics of thioflavin T (ThT) binding was monitored by bottom reading of fluorescence intensity in a POLARstar microplate reader (BMG Labtech) as described [51]. Measurements were performed using 450 nm excitation and 480 nm emission filters, 0.20 ml samples and 15 µM ThT concentration. The measurement program consisted of 10 flashes reading every 10 min with 1-min of orbital 1-mm diameter shaking at 100 rpm with the temperature controller set at 30°C. All measurements were done in triplicate and the experiments were repeated at least twice using two different peptide batches. When required, the lag-phase was determined as described [61].

### PAGE analysis

Peptide samples before and after 80 h of aggregation were removed and diluted 1∶1 in β-mercaptoethanol-free Laemmli buffer and, omotting the thermal denaturation step, loaded in BioRad precastTGX-gels. After silver staining, gel images were captured and analyzed using the Molecular Imager ChemiDoc™ XRS+ Imaging system and ImageLab 3.0.1 (beta2) software (BioRad).

### Mass spectrometry analysis

Aliquots of the peptide solutions before and after aggregation were removed, treated with HFIP for aggregate disruption and analyzed using α-hydroxy-cinnamic acid matrix and a MALDI FT-ICR 930-MS (Varian) instrument operating at 7 T and 10^−9^ Torr and with OMEGA software.

### Atomic force microscopy (AFM)

Ten µl-samples of peptide solution after 70 h incubation were diluted to 2 µM with ddH_2_O and applied onto freshly cleaved mica surfaces to adhere for 15 min. After washing with ddH_2_O, samples were dried with N_2_. AFM imaging was then performed using a PicoSPM™ (Molecular Imaging, Phoenix, AZ), operating the AFM scanner in acoustic alternating current mode with Si,N-ACT type cantilevers (ScienTec) with a tip radius <10 nm and a spring constant of 25–75 N/m [51]. The images (1×1 µm scans) were collected at a scan rate of 1 line per second and analyzed using WSxM 5.0 Nanotec software.

### Circular dichroism (CD) spectroscopy

CD spectra were recorded in the far-UV region with a Jasco J-810 spectropolarimeter in continuous scan mode (250−190 nm) and a 0.1 cm path length quartz cuvette (Hellma) as described previously [51].

### Citotoxicity assays

Mice were obtained from the Centro de Biología Molecular and treated following the guidelines of Council of Europe Convention ETS123, recently revised as indicated in the Directive 86/609/EEC. Animal experiments were performed under protocols (P22/P23) approved by the Centro de Biología Molecular Severo Ochoa Institutional Animal Care and Utilization Committee (CEEA-CBM, Madrid, Spain). Primary cortical neurons were obtained from the cerebral cortex of C57B16 E18 rat embryos, by enzymatic dissociation with papain (Worthington Biochemical) in EBSS for 45 min at 37°C. Cells were resuspended in Neurobasal medium with 2% B27, 0.25% 200 mM Gln, 1% Glutamax and 1% penicillin/streptomycin and seeded on cover slips pre-coated with poly-D-Lys (10 µg/ml). Two days later, 5 µM Ara-C was added to the medium. Seven days later, peptides preincubated in PBS were added at a final concentration of 25 µM. After 48 h incubation at 37°C, neuronal cell death was determined using the LIVE/DEAD kit (Invitrogen) for mammalian cells. Live cells (stained with calcein-AM) and dead cells (stained with red-fluorescent ethidium homodimer-1) were counted and the percentage of dead cells calculated.

## Supporting Information

Supporting Information S1 (DOCX)
