# Supplementary material for: Selenomethionine Incorporation into Amyloid Sequences Regulates Fibrillogenesis and Toxicity
Source: PLoS One. 2011 Nov 22;6(11):e27999. doi: 10.1371/journal.pone.0027999 (PMC3222675; doi:10.1371/journal.pone.0027999)
Supplement: Supporting Information S1 — (DOCX) [file pone.0027999.s001.docx]

**Supporting Information**

**1. Materials and Methods for [SeM^35^]Aβ(1-40)**

**Abbreviations:** DCM, dichloromethane; DIEA, *N*,*N*-diisopropylethylamine; DIPCDI, diiso­propylcarbodiimide; DMF, N,N-dimethylformamide Fmoc, 9-fluorenylmethoxycarbonyl; HBTU, 2-(1*H*-benzotriazol-1-yl)-1,1,3,3-tetramethyluronium hexafluorophosphate; HPLC, high performance liquid chromatography; MALDI-TOF, matrix-assisted laser desorption ionization time-of-flight; MS, mass spectrometry; SeM, selenomethionine; SPPS, solid phase peptide synthesis; TFA, trifluoroacetic acid; TIS, triisopropylsilane.

**Chemicals.** Fmoc-protected amino acids were obtained from Senn Chemicals (Dielsdorf, Switzerland). Fmoc-L-SeM-OH was from AnaSpec (San Jose, CA, USA), and Boc-Ser(Fmoc-Gly)-OH from Novabiochem (Läufelfingen, Switzerland). Fmoc-Rink-amide ChemMatrix resin was from Matrix Innovation (Montreal, Canada). HPLC-grade acetonitrile, and peptide synthesis-grade DMF, DCM, DIEA and TFA were from Carlo Erba-SdS (Sabadell, Spain). All other reagents were of the highest quality commercially available.

**26-O-isoacyl-[SeM^35^]Aβ(1-40).** The synthetic approach is illustrated on **Figure S1**. Automated synthesis was performed in an ABI433 synthesizer (Applied Biosystems) running FastMoc protocols at 0.08 mmol scale on Fmoc-Rink-amide ChemMatrix resin [1]. 10-fold molar excess of Fmoc-L-amino acids and HBTU/HOBt, in the presence of 20-fold molar excess of DIEA, were used for coupling, with DMF as solvent. Side-chains were protected with the TFA-labile groups t-butyl (Asp, Glu, Ser, Tyr), t-butyloxycarbonyl (Lys), 2,2,4,6,7-pentamethyldihydrobenzofuran-5-sulfonyl (Arg), and trityl (Asn, Gln, His). Boc-Ser(Fmoc-Gly)-OH was coupled in the manual mode (polypropylene syringe with a porous polyethylene disk) in 4-fold molar excess in the presence of an equivalent amount of DIPCDI and HOBt in DMF. After synthesis completion, the protected peptide resin was N-deblocked with piperidine/DMF (20% v/v) prior to full deprotection and cleavage with TFA/H_2_O/TIS (95:2.5:2.5 v/v, 90 min, 25 ºC). The peptide was precipitated by addition of chilled diethyl ether, taken up in aqueous acetic acid (10% v/v) and lyophilized.

Analytical RP-HPLC was done on a C_8_ column (4.6×50 mm, 3 μm, Phenomenex) in a LC-2010A system (Shimadzu). Solvents A and B were 0.045% and 0.036% (v/v) TFA in H_2_O and MeCN, respectively. A linear 20-50% gradient of B into A over 15 min at 1mL/min flow rate was used for elution, with UV detection at 220 nm. Preparative HPLC was done on a C_8_ column (10×250 mm, 10 μm, Phenomenex) in a Shimadzu LC-8A system. Solvents A and B were 0.1% TFA (v/v) in H_2_O and MeCN, respectively. A linear 20-35% gradient of B into A over 30 min was used for elution, at 5 mL/min flow rate, with UV detection at 220 nm. Fractions of satisfactory purity (>95%) by analytical HPLC were pooled and lyophilized (**Figure S2**). The purified peptide was satisfactorily checked for identity by MALDI-TOF MS in a Voyager DE-STR instrument (Applied Biosystems), in the reflector mode, with α-hydroxy-cinnamic acid matrix: calculated MW for C_194_H_296_N_54_O_57_Se_1_= 4374.1089 (monoisotopic), found: [M+H^+^] 4375.45.

**[SeM^35^]Aβ(1-40).** The target product was obtained in an O-N acyl shift reaction [2,3] upon incubation of purified 26-O-isoacyl-[SeM^35^]Aβ(1-40) in PBS, pH 7.4 for 4 h at 37°C. The peptide precipitate was centrifuged and washed with water. Progress of the rearrangement reaction is shown in Figure S3. The end product was satisfactorily characterized by MALDI-TOF MS, as above. Calculated MW for C_194_H_296_N_54_O_57_Se_1_= 4375.7718 (monoisotopic), m/z found: [M+H^+^]: 4375.6

**2. Materials and Methods for HuPrP(106-140) and analogs**

**Abbreviations:** DCM, dichloromethane; DIEA, *N*,*N*-diisopropylethylamine; DMF, N,N-dimethylformamide; Fmoc, 9-fluorenylmethoxycarbonyl; TBTU, 2-(1*H*-benzotriazole-1-yl)-1,1,3,3-tetramethyluronium hexafluorophosphate; HPLC, high performance liquid chromatography; MALDI-TOF, matrix-assisted laser desorption ionization time-of-flight; MS, mass spectrometry; SeM, selenomethionine; SPPS, solid phase peptide synthesis; TFA, trifluoroacetic acid; TIS, triisopropylsilane.

**Chemicals.** Fmoc-protected amino acids and linkers were from Luxembourg Industries (Tel-Aviv, Israel), Neosystem (Strasbourg, France), Calbiochem-Novabiochem (Laüfelfingen, Switzerland), Bachem AG (Bubendorf, Switzerland) and Iris Biotech (Marktredwitz, Germany). 4-Aminomethyl ChemMatrix resin was from Matrix Innovation (Montreal, Canada). DIEA and TIS were from Sigma (St. Louis, MO). TBTU was from Iris Biotech. Solvents for peptide synthesis and HPLC were from Carlo Erba-SdS (Sabadell, Spain). TFA was from Fluorochem Ltd (Derbyshire, UK). All other chemicals were purchased from Sigma-Aldrich at the highest quality available.

**Peptide synthesis and characterization.** Peptides were synthesized at 100 µmol scale [4]. Fmoc-Rink amide linker was manually coupled in a 4-fold molar excess to 4-aminomethyl ChemMatrix resin (0.57 mmol/g) in the presence of TBTU (4-fold excess) and DIEA (8-fold excess) in 3 mL of DMF, with 5 min preactivation followed by 90 min coupling to the resin. Completeness of coupling was estimated by the Kaiser test [5]. Peptides were assembled on this resin by automated Fmoc synthesis protocols (see above) run on a Liberty-12-channel synthesizer (Matthews, NC). Briefly, Fmoc groups were removed with piperidine/DMF (+0.1M HOBt; 1:4) using a short (37 W, 79 ºC, 30 s) followed by a long cycle (37 W, 79 ºC, 180 s). After DMF and DCM washings, coupling was carried out with a 0.2 M solution of Fmoc-amino acid, in the presence of 0.5 M TBTU and 2 M DIEA at 21 W, 79 ºC for 5 min. Fmoc-Arg(Pbf) required an additional coupling step consisting of a long cycle at 0 W, 25 ºC for 25 min followed by two short cycles at 21W, 75 ºC for 5 min. For Fmoc-His(Trt), deprotection was at 50 ºC to avoid racemization, and a specific coupling cycle, again to avoid racemization, was used, consisting of an initial step at 0 W, 50 ºC for 4 min followed by another at 15 W, 50 ºC for 5 min. After synthesis completion, the N-deblocked peptide resins were side-chain deprotected and cleaved from the resin using TFA/H_2_O/EDT/TIS (94:2.5:2.5:1, 3 h) with mild orbital shaking. The peptides were precipitated by addition of chilled diethyl ether, taken up in aqueous acetic acid (10% v/v) and lyophilized.

Peptides were analyzed by HPLC on a Sunfire C_18_ column (4.6×100 mm, 3.5 μm) in a Waters 2695 system equipped with a 996 PDA and Empower software. Solvents A and B were 0.045% and 0.036% (v/v) TFA in H_2_O and MeCN, respectively. Linear 0-100% gradients of B into A over 8 min at 1 mL/min flow rate were used for elution, with UV detection at 220 nm. Preparative HPLC was done on a Symmetry C_18_ column (30×100 mm, 5 μm, Waters) in a Waters 600 system. Solvents A and B were 0.1% TFA (v/v) in H_2_O and 0.05% TFA (v/v) in MeCN, respectively. Linear 10-60% gradients of B into A over 30 min were used for elution, at 10 mL/min flow rate, with UV detection at 220 nm. HPLC traces and purities of the purified peptides are shown in **Figure S3** and **Table S1**, respectively. Peptides were satisfactorily checked for identity by mass spectrometry on MALDI-TOF/TOF 4700 (Applied Biosystems) and (for higher resolution) LTQ-FT Ultra (Thermo Scientific) systems. Mass data are given in **Table S1**. Peptide quantification was done by amino acid analysis with the Waters AccQ-Tag system.

**3. References**

1. Fields GB, Noble RL. (1990) Solid phase peptide synthesis utilizing 9-fluorenylmethoxycarbonyl amino acids. Int J Pept Protein Res 35: 161-214

2. Sohma Y, Kiso Y. (2006) Click peptides"--chemical biology-oriented synthesis of Alzheimer's disease-related amyloid beta peptide (abeta) analogues based on the "O-acyl isopeptide method. Chem. Biochem. 7: 1549-1557

3. Sohma Y, Yoshiya T, Taniguchi A, Kimura T, Hayashi Y, et al. (2007) Development of O-acyl isopeptide method. Biopolymers 88: 253-262.

4. Grillo-Bosch D, Rabanal F, Giralt E. (2011) Improved Fmoc-based solid-phase synthesis of homologous peptide fragments of human and mouse prion proteins. J. Pept. Sci. 17: 32-38.

5.Kaiser E, Colescott RL, Bossinger CD, Cook PI. (1970) Color test for detection of free terminal amino groups in the solid-phase síntesis of peptides. Anal. Biochem. 34: 595-598.

**Table S1.** HPLC and mass spectrometric characterization of HuPrP peptides

| **Peptide** | **Purity** | **Theo. Mass** | **Mass [M+ H^+^]** |
| --- | --- | --- | --- |
| all-M | 98.3% | 3423.751 | 3424.725 |
| all-V | 99.2% | 3295.862 | 3296.932 |
| [SeM^109^]HuPrP(106-140) | 98.4% | 3375.779 | 3376.470 |
| [SeM^112^]HuPrP(106-140) | 96.5% | 3375.779 | 3376.854 |
| [SeM^129^]HuPrP(106-140) | 97.8% | 3375.779 | 3376.927 |
| [SeM^134^]HuPrP(106-140) | 97.5% | 3375.779 | 3376.882 |

**4. Supporting Information Legends**

**Figure S1:** Synthesis of [SeM^35^]Aβ(1-40) by the O-acyl isopeptide method: (i) deprotection with 20% piperidine in DMF; (ii) coupling of Fmoc-L-AA/HBTU/HOBt/DIEA (10/10/10/20 eq); (iii) coupling of Boc-L-Ser(Fmoc-Gly)-OH/DIPCDI/HOBt (4/4/4 eq); (iv) cleavage: TFA/H_2_O/TIS (95:2.5:2.5, v/v); (v) HPLC purification; (vi) rearrangement: PBS, pH 7.4, 37°C, 3 h; (vii) H_2_O washes. Sequences (X=SeM): Aβ(27-40) = NKGAIIGLXVGGVV; Aβ(1-24) = DAEFRHDSGYEVHHQKLVFFAEDV

**Figure S2:** Progress of the O→N shift reaction of 26-O-isoacyl-[SeM^35^]Aβ(1-40) to [SeM^35^]Aβ(1-40) by incubation in PBS at pH 7.4, 37 ºC, for 4 h. HPLC conditions as described in text above.

**Figure S3:** HPLC characterization of (left to right, top to bottom) all-M, [SeM109]HuPrP(106-140), [SeM112]HuPrP(106-140), [SeM129]HuPrP(106-140), [SeM134]HuPrP(106-140) and all-V peptides. HPLC conditions in text above.

**Figure S1**.


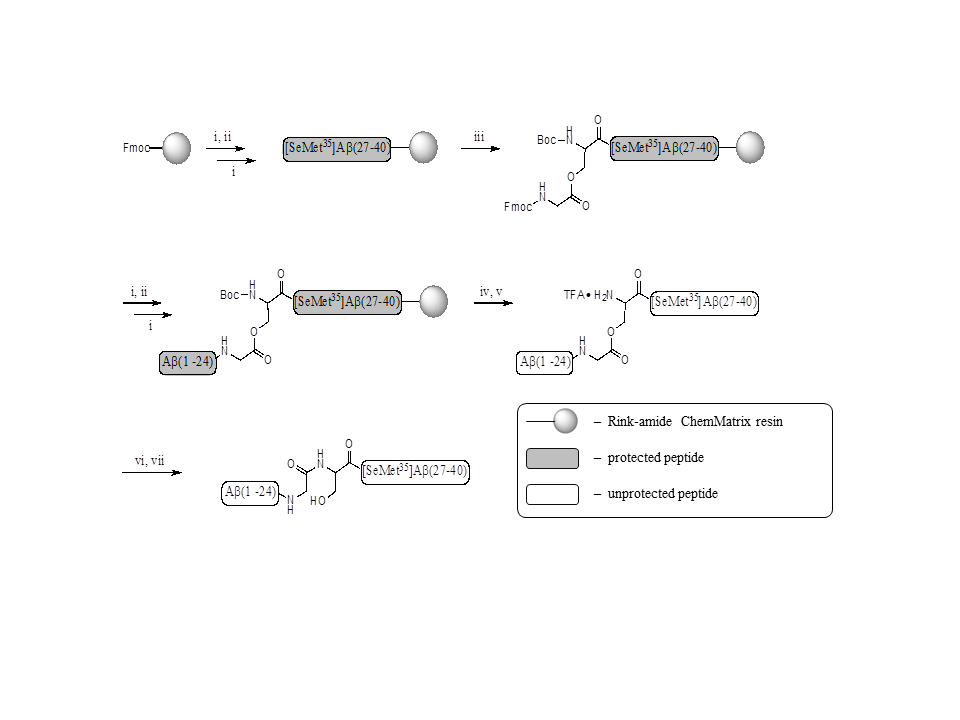


**Figure S2.**

**Figure S3.**
